# 1,1′-Dimethyl-1,1′-(butane-1,4-di­yl)dipyrrolidinium dibromide methanol disolvate

**DOI:** 10.1107/S1600536808000172

**Published:** 2008-01-11

**Authors:** Yu-Lin Yang, Wen-Jiu Wang, Wen-Hui Li, Rui-Qing Fan

**Affiliations:** a Department of Applied Chemistry, Harbin Institute of Technology, Harbin 150001, People’s Republic of China; b College of Materials Science and Engineering, Harbin University of Science & Technology, Harbin 150040, People’s Republic of China

## Abstract

In the title compound, C_14_H_30_N_2_^2+^·2Br^−^·2CH_3_OH, two terminal C atoms of the butane chain are connected to two N atoms of the 1-methyl­pyrollidines, forming a linear diquaternary ammonium cation. The cation lies across a centre of inversion located between the two central C atoms of the butane chain. The asymmetric unit therefore comprises one half-cation, a bromide anion and a methanol solvent mol­ecule. In the crystal structure, the bromide anions are linked to the methanol solvent mol­ecules by O—H⋯Br hydrogen bonds.

## Related literature

For information on the use of organic amines in zeolite synthesis, see: Gramm *et al.* (2006 ▶); Hong *et al.* (2007 ▶). For a previous synthesis of the title compound, see: Hong *et al.* (2004 ▶).
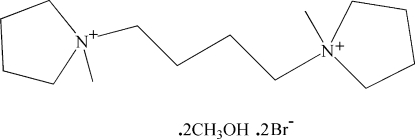


## Experimental

### 

#### Crystal data

C_14_H_30_N_2_^2+^·2Br^−^·2CH_4_O
*M* *_r_* = 450.30
Monoclinic, 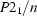

*a* = 6.4919 (7) Å
*b* = 12.4861 (13) Å
*c* = 12.9683 (13) Å
β = 90.748 (2)°
*V* = 1051.10 (19) Å^3^
*Z* = 2
Mo *K*α radiation
μ = 3.87 mm^−1^
*T* = 193 (2) K
0.30 × 0.25 × 0.24 mm

#### Data collection

Bruker SMART APEX CCD area-detector diffractometer
Absorption correction: multi-scan (*SADABS*; Bruker, 2000 ▶) *T*_min_ = 0.390, *T*_max_ = 0.457 (expected range = 0.337–0.396)
5618 measured reflections
2013 independent reflections
1681 reflections with *I* > 2σ(*I*)
*R*_int_ = 0.022

#### Refinement

*R*[*F* ^2^ > 2σ(*F* ^2^)] = 0.027
*wR*(*F* ^2^) = 0.067
*S* = 1.09
2013 reflections
102 parameters
H-atom parameters constrained
Δρ_max_ = 0.42 e Å^−3^
Δρ_min_ = −0.20 e Å^−3^

### 

Data collection: *SMART* (Bruker, 2000 ▶); cell refinement: *SAINT* (Bruker, 2000 ▶); data reduction: *SAINT*; program(s) used to solve structure: *SHELXS97* (Sheldrick, 2008 ▶); program(s) used to refine structure: *SHELXL97* (Sheldrick, 2008 ▶); molecular graphics: *SHELXTL* (Sheldrick, 1997 ▶); software used to prepare material for publication: *SHELXTL*.

## Supplementary Material

Crystal structure: contains datablocks global, I. DOI: 10.1107/S1600536808000172/sj2458sup1.cif

Structure factors: contains datablocks I. DOI: 10.1107/S1600536808000172/sj2458Isup2.hkl

Additional supplementary materials:  crystallographic information; 3D view; checkCIF report

## Figures and Tables

**Table 1:** Hydrogen-bond geometry (Å, °)

*D*—H⋯*A*	*D*—H	H⋯*A*	*D*⋯*A*	*D*—H⋯*A*
O1—H1⋯Br1	0.82	2.43	3.2453 (18)	172

**Fig. 1.**
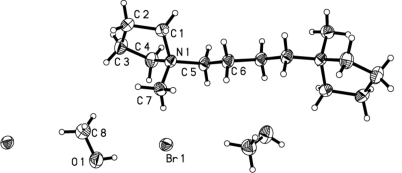
View of the molecule of (I) showing the atom-labelling scheme. Displacement ellipsoids are drawn at the 50% probability level. Labelled atoms are related to unlabelled atoms by the symmetry operation-x + 1,-y,-z + 2.

**Fig. 2.**
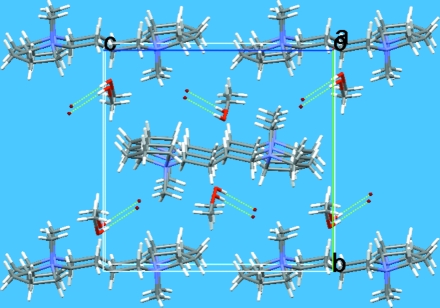
The molecular packing of (I) with hydrogen bonds drawn as dashed lines.

## supplementary crystallographic information

### Comment

The use of zeolites as catalysts or catalyst supports is now widely applied in petrochemical and fine chemical processes. The synthesis of zeolites involves the addition of organic amines and it is proposed that in most cases, the amine acts as a structure-directing agent, helping to shape the framework of the structure. TNU-9 is a complex zeolite (Gramm *et al.*, 2006) and the title compound, (I), is used as structure-directing agent in the synthesis of the TNU-9 zeolite (Hong *et al.*, 2007). In this paper, we report a modified synthesis and the crystal structure of (I), Fig 1.

The structure of (I) consists of a linear diquaternary ammonium cation, two bromide anions and two methanol solvate molecules. The cation lies about an inversion centre at the centroid of the C6—C6A bond in the butane chain. The terminal carbon atoms of the butane are connected to the N atoms of the 1-methylpyrolidines, forming a linear diquaternary ammonium cation. In the crystal structure Br^-^ anions are linked to methanol molecules by O1—H1···Br1 hydrogen bonds that stabilize the structure (Fig 2, Table 1).

### Experimental

(I) was prepared by refluxing 1,4-dibromobutane (1 mmol, 99%, Arcos) with an excess of 1-methylpyrrolidine (3 mmol, 97%, Arcos) 24 h in acetone (150 ml, 99%, Arcos), in a modification of the previously reported procedure (Hong *et al.*, 2004). The excess amine was removed by extraction with acetone, and recrystallizations were performed in a methanol-diethylether mixtures (2:1).

### Refinement

H atoms were positioned geometrically with O—H = 0.82 and C—H = 0.96–0.97 Å, and allowed to ride on their parent atoms with *U*_iso_(H) = 1.2 *U*_eq_(C) for CH_2_ groups, and 1.5 *U*_eq_(C,*O*) for the –OH and –CH_3_ groups.

### Crystal data

**Table:**

C_14_H_30_N_2_^2+^·2Br^–^·2CH_4_O	*F*_000_ = 468
*M**_r_* = 450.30	*D*_x_ = 1.423 Mg m^−^^3^
Monoclinic, *P*2_1_/*n*	Mo *K*α radiation λ = 0.71073 Å
Hall symbol: -P 2yn	Cell parameters from 5618 reflections
*a* = 6.4919 (7) Å	θ = 2.3–26.0º
*b* = 12.4861 (13) Å	µ = 3.87 mm^−^^1^
*c* = 12.9683 (13) Å	*T* = 193 (2) K
β = 90.748 (2)º	Block, colorless
*V* = 1051.10 (19) Å^3^	0.30 × 0.25 × 0.24 mm
*Z* = 2	

### Data collection

**Table:**

Bruker SMART APEX CCD area-detector diffractometer	2013 independent reflections
Radiation source: fine-focus sealed tube	1681 reflections with *I* > 2σ(*I*)
Monochromator: graphite	*R*_int_ = 0.022
*T* = 193(2) K	θ_max_ = 26.0º
φ and ω scans	θ_min_ = 2.3º
Absorption correction: multi-scan(SADABS; Bruker, 2000)	*h* = −5→8
*T*_min_ = 0.390, *T*_max_ = 0.457	*k* = −14→15
5618 measured reflections	*l* = −15→16

### Refinement

**Table:**

Refinement on *F*^2^	Secondary atom site location: difference Fourier map
Least-squares matrix: full	Hydrogen site location: inferred from neighbouring sites
*R*[*F*^2^ > 2σ(*F*^2^)] = 0.027	H-atom parameters constrained
*wR*(*F*^2^) = 0.067	*w* = 1/[σ^2^(*F*_o_^2^) + (0.0335*P*)^2^ + 0.1193*P*] where *P* = (*F*_o_^2^ + 2*F*_c_^2^)/3
*S* = 1.09	(Δ/σ)_max_ < 0.001
2013 reflections	Δρ_max_ = 0.42 e Å^−^^3^
102 parameters	Δρ_min_ = −0.20 e Å^−^^3^
Primary atom site location: structure-invariant direct methods	Extinction correction: none

### Special details

**Table:**

Geometry. All e.s.d.'s (except the e.s.d. in the dihedral angle between two l.s. planes) are estimated using the full covariance matrix. The cell e.s.d.'s are taken into account individually in the estimation of e.s.d.'s in distances, angles and torsion angles; correlations between e.s.d.'s in cell parameters are only used when they are defined by crystal symmetry. An approximate (isotropic) treatment of cell e.s.d.'s is used for estimating e.s.d.'s involving l.s. planes.
Refinement. Refinement of *F*^2^ against ALL reflections. The weighted *R*-factor *wR* and goodness of fit *S* are based on *F*^2^, conventional *R*-factors *R* are based on *F*, with *F* set to zero for negative *F*^2^. The threshold expression of *F*^2^ > σ(*F*^2^) is used only for calculating *R*-factors(gt) *etc*. and is not relevant to the choice of reflections for refinement. *R*-factors based on *F*^2^ are statistically about twice as large as those based on *F*, and *R*- factors based on ALL data will be even larger.

### Fractional atomic coordinates and isotropic or equivalent isotropic displacement parameters (Å^2^)

**Table:**

	*x*	*y*	*z*	*U*_iso_*/*U*_eq_	
Br1	0.22798 (4)	0.222545 (19)	0.645609 (19)	0.03899 (11)	
N1	0.6827 (3)	0.01906 (14)	0.77454 (14)	0.0279 (4)	
C1	0.8754 (4)	−0.04680 (19)	0.79320 (18)	0.0380 (6)	
H1A	0.8411	−0.1171	0.8198	0.046*	
H1B	0.9671	−0.0113	0.8420	0.046*	
C2	0.9755 (4)	−0.0559 (2)	0.68752 (18)	0.0443 (7)	
H2A	1.0286	−0.1276	0.6772	0.053*	
H2B	1.0884	−0.0054	0.6820	0.053*	
C3	0.8074 (4)	−0.0306 (2)	0.60735 (19)	0.0467 (7)	
H3A	0.8386	0.0351	0.5708	0.056*	
H3B	0.7939	−0.0884	0.5577	0.056*	
C4	0.6110 (4)	−0.0184 (2)	0.66926 (17)	0.0385 (6)	
H4A	0.5197	0.0339	0.6375	0.046*	
H4B	0.5390	−0.0862	0.6741	0.046*	
C5	0.5187 (3)	−0.00229 (18)	0.85296 (17)	0.0316 (5)	
H5A	0.4768	−0.0767	0.8475	0.038*	
H5B	0.3994	0.0415	0.8364	0.038*	
C6	0.5842 (3)	0.01998 (18)	0.96372 (16)	0.0319 (5)	
H6A	0.6052	0.0962	0.9734	0.038*	
H6B	0.7129	−0.0164	0.9792	0.038*	
C7	0.7340 (4)	0.13607 (17)	0.77065 (18)	0.0344 (5)	
H7A	0.7800	0.1595	0.8376	0.052*	
H7B	0.6137	0.1759	0.7503	0.052*	
H7C	0.8412	0.1477	0.7216	0.052*	
O1	0.5324 (3)	0.32947 (15)	0.47786 (14)	0.0510 (5)	
H1	0.4456	0.3057	0.5171	0.076*	
C8	0.6858 (4)	0.2517 (2)	0.4620 (2)	0.0478 (7)	
H8A	0.7952	0.2615	0.5116	0.072*	
H8B	0.7392	0.2590	0.3937	0.072*	
H8C	0.6275	0.1816	0.4700	0.072*	

### Atomic displacement parameters (Å^2^)

**Table:**

	*U*^11^	*U*^22^	*U*^33^	*U*^12^	*U*^13^	*U*^23^
Br1	0.03054 (16)	0.03698 (16)	0.04954 (18)	0.00222 (11)	0.00417 (11)	0.00310 (11)
N1	0.0241 (10)	0.0302 (9)	0.0296 (10)	0.0005 (8)	0.0036 (8)	−0.0010 (8)
C1	0.0336 (13)	0.0414 (14)	0.0390 (14)	0.0110 (11)	0.0052 (11)	0.0050 (11)
C2	0.0438 (16)	0.0413 (14)	0.0482 (16)	0.0130 (12)	0.0163 (13)	0.0038 (12)
C3	0.0487 (16)	0.0560 (17)	0.0359 (14)	0.0039 (14)	0.0113 (13)	−0.0063 (12)
C4	0.0396 (14)	0.0453 (14)	0.0307 (12)	−0.0027 (12)	−0.0011 (11)	−0.0059 (11)
C5	0.0243 (12)	0.0349 (12)	0.0359 (13)	−0.0060 (10)	0.0079 (10)	−0.0032 (10)
C6	0.0265 (12)	0.0337 (12)	0.0356 (13)	−0.0031 (10)	0.0072 (10)	−0.0019 (10)
C7	0.0318 (13)	0.0308 (12)	0.0406 (14)	−0.0050 (10)	0.0041 (11)	0.0040 (10)
O1	0.0450 (12)	0.0590 (12)	0.0492 (12)	0.0083 (10)	0.0092 (9)	0.0080 (9)
C8	0.0447 (17)	0.0497 (15)	0.0492 (17)	0.0009 (13)	0.0048 (14)	−0.0040 (12)

### Geometric parameters (Å, °)

**Table:**

N1—C7	1.500 (3)	C5—C6	1.518 (3)
N1—C5	1.506 (3)	C5—H5A	0.9700
N1—C4	1.511 (3)	C5—H5B	0.9700
N1—C1	1.514 (3)	C6—C6^i^	1.535 (4)
C1—C2	1.528 (3)	C6—H6A	0.9700
C1—H1A	0.9700	C6—H6B	0.9700
C1—H1B	0.9700	C7—H7A	0.9600
C2—C3	1.530 (4)	C7—H7B	0.9600
C2—H2A	0.9700	C7—H7C	0.9600
C2—H2B	0.9700	O1—C8	1.408 (3)
C3—C4	1.523 (3)	O1—H1	0.8200
C3—H3A	0.9700	C8—H8A	0.9600
C3—H3B	0.9700	C8—H8B	0.9600
C4—H4A	0.9700	C8—H8C	0.9600
C4—H4B	0.9700		
			
C7—N1—C5	110.76 (17)	C3—C4—H4B	110.8
C7—N1—C4	109.70 (18)	H4A—C4—H4B	108.8
C5—N1—C4	110.08 (17)	N1—C5—C6	114.51 (17)
C7—N1—C1	110.55 (18)	N1—C5—H5A	108.6
C5—N1—C1	112.72 (17)	C6—C5—H5A	108.6
C4—N1—C1	102.75 (17)	N1—C5—H5B	108.6
N1—C1—C2	104.89 (18)	C6—C5—H5B	108.6
N1—C1—H1A	110.8	H5A—C5—H5B	107.6
C2—C1—H1A	110.8	C5—C6—C6^i^	109.1 (2)
N1—C1—H1B	110.8	C5—C6—H6A	109.9
C2—C1—H1B	110.8	C6^i^—C6—H6A	109.9
H1A—C1—H1B	108.8	C5—C6—H6B	109.9
C1—C2—C3	106.6 (2)	C6^i^—C6—H6B	109.9
C1—C2—H2A	110.4	H6A—C6—H6B	108.3
C3—C2—H2A	110.4	N1—C7—H7A	109.5
C1—C2—H2B	110.4	N1—C7—H7B	109.5
C3—C2—H2B	110.4	H7A—C7—H7B	109.5
H2A—C2—H2B	108.6	N1—C7—H7C	109.5
C4—C3—C2	104.9 (2)	H7A—C7—H7C	109.5
C4—C3—H3A	110.8	H7B—C7—H7C	109.5
C2—C3—H3A	110.8	C8—O1—H1	109.5
C4—C3—H3B	110.8	O1—C8—H8A	109.5
C2—C3—H3B	110.8	O1—C8—H8B	109.5
H3A—C3—H3B	108.8	H8A—C8—H8B	109.5
N1—C4—C3	104.9 (2)	O1—C8—H8C	109.5
N1—C4—H4A	110.8	H8A—C8—H8C	109.5
C3—C4—H4A	110.8	H8B—C8—H8C	109.5
N1—C4—H4B	110.8		

Symmetry codes: (i) −*x*+1, −*y*, −*z*+2.

### Hydrogen-bond geometry (Å, °)

**Table:**

*D*—H···*A*	*D*—H	H···*A*	*D*···*A*	*D*—H···*A*
O1—H1···Br1	0.82	2.43	3.2453 (18)	172
